# The effect of biphasic cuirass ventilation on peripheral lymphatic function in young adults

**DOI:** 10.14814/phy2.71161

**Published:** 2026-10-30

**Authors:** C. Noah Nilsson, Lene Thorup, Benjamin Kelly, Jakob Friis Schmidt, Michael Seltz, Vibeke Hjortdal

**Affiliations:** ^1^ Department of Cardiothoracic Surgery Copenhagen University Hospital Copenhagen Denmark; ^2^ Department of Anesthesiology, Centre of Head and Orthopaedics Copenhagen University Hospital Copenhagen Denmark

**Keywords:** biphasic cuirass ventilation, edema, fluid homeostasis, lymphatic system, negative pressure ventilation, respiratory pump

## Abstract

Positive pressure ventilation increases the intrathoracic pressure and the central venous pressure, impeding venous return to the heart. Likewise, both central and peripheral lymphatic function is haltered during positive pressure ventilation, presenting as a possible explanation for fluid retention and edema in post‐operative and intensive care unit patients. We aimed to examine the effects of biphasic cuirass ventilation on the peripheral lymphatic system. Peripheral lymphatic function was examined in twenty healthy, young adults during spontaneous breathing and during biphasic cuirass ventilation. The peripheral lymphatic function was assessed with near‐infrared fluorescence imaging, and the microvascular circulation was assessed with strain gauge plethysmography. Mean peripheral lymphatic contraction frequency (mean ± SD) was 1.46 ± 0.73/min at baseline and 1.43 ± 0.76/min during biphasic cuirass ventilation. There was no significant difference (mean difference = 0.03, 95% CI: −0.23–0.29, *p* = 0.81, *n* = 19). Capillary filtration rates did not differ between baseline and biphasic cuirass ventilation across all pressure levels (*F*(1,18) = 1.13, *p* = 0.30). Likewise, there were no significant differences in isovolumetric pressures (mean difference = −2.61, 95% CI: −8.92–3.71, *p* = 0.39, *n* = 15), or capillary filtration coefficients (mean difference = 1.21 × 10^−4^, 95% CI: −4,87 × 10^−4^ – 7.29 × 10^−4^, *p* = 0.68, *n* = 19). We observed no changes in peripheral lymphatic activity in awake, healthy, young adults during BCV.

## 1 INTRODUCTION

Every day, the lymphatic system absorbs between 8 and 12 liters of interstitial fluid through the lymphatic capillaries. Around half re‐enters the systemic circulation via the great veins, and the other half is returned via the lymph nodes (Adair & Guyton, 1983; Knox & Pflug, 1983; Levick & Michel, 2010; Renkin, 1986). Transportation of lymph is driven by the active pumping of lymphatic vessels along with the passive forces of interstitial fluid accumulation and extrinsic tissue pressures acting on the vessels while intraluminal valves ensure the unidirectional flow of lymph (Breslin et al., 2018). The thoracic duct receives the majority of the lymph from collecting lymphatic vessels and drains through the ostial valve into the junction of the left subclavian vein and the left internal jugular vein (Brotons et al., 2012; El Zawahry et al., 1983). The lymphatic drainage is thus dependent on the pressure gradient between the pressure in the thoracic duct and the central venous pressure (CVP), both of which are sensitive to the changes in intrathoracic pressure during respiration. The increased intrathoracic pressure observed during positive pressure ventilation (PPV) with positive‐end‐expiratory pressure (PEEP) increases both CVP and thoracic duct pressure (Kelly et al., 2022), resulting in impaired venous return (Corp et al., 2021; Umbrello et al., 2023) and impaired thoracic duct outflow (Khan et al., 2023). Recently, our group also showed that peripheral lymphatic activity is decreased during PPV (Thorup et al., 2025), suggesting that the disruptive effects on the lymphatic system seen during PPV may contribute to fluid retention and edema in post‐operative and intensive care unit (ICU) patients, a significant source of morbidity and mortality in ICU patients (Claure‐Del Granado & Mehta, 2016; Messmer et al., 2020). One could therefore hypothesize that decreasing the intrathoracic pressure—and in turn the thoracic duct pressure and CVP—would have the opposite effect and improve lymphatic drainage. Negative pressure ventilation (NPV), including biphasic cuirass ventilation (BCV), achieved with an extra‐thoracic cuirass shell, has demonstrated beneficial hemodynamic effects, primarily driven by an increase in preload, in both children and adults, and on both the right and the left ventricle (McBride et al., 2012a, 2012b; Okada et al., 2018; Scholz et al., 1997; Shekerdemian et al., 1996; Shekerdemian, Bush, Lincoln, et al., 1997; Shekerdemian, Bush, Shore, et al., 1997; Toida et al., 2007). However, the effects of BCV on the lymphatic system remain unknown. To investigate this, we performed a systematic evaluation of the effect of BCV on the peripheral lymphatic system in healthy, young adults. The findings from this investigator‐initiated, experimental, interventional study could guide us to the optimal balance between intrathoracic pressure and lymphatic transport, limiting and ameliorating fluid retention and edema in post‐operative and ICU patients.

## 2 METHODS

### 2.1 Study registration

This study was approved by the Scientific Ethics Committees for the Capital Region of Denmark (H‐23031273) and was in accordance with the Declaration of Helsinki. All participants gave written informed consent upon enrollment.

### 2.2 Study population

Twenty cardio‐pulmonary and lymphatically healthy, young adults were recruited with posters and by word of mouth at Copenhagen University Hospital and University of Copenhagen.

#### 2.2.1 Inclusion criteria

Ages of ≥18 and ≤40 years. BMI ≤30.

#### 2.2.2 Exclusion criteria

Individuals with a history of lymphatic or systemic diseases: cancer, heart disease, pulmonary disease, lymphatic disease, chronic and long‐term infectious disease, lymphedema, edema, and/or venous disease. Along with individuals with previous surgical interventions in the thorax.

### 2.3 Biphasic cuirass ventilation

Cuirass ventilation works by placing a shell, the cuirass, with an airtight seal on the anterior chest and upper abdomen. Subsequently, the cuirass is attached to a power unit that actively controls inspiration and expiration by controlling the pressure inside the cuirass (Hayek, United Hayek Medical, London, United Kingdom). Attachment and function of the cuirass is demonstrated in the following video: https://airwaymanagement.dk/attaching_cuirass_and_ventilating. BCV “Control mode” was chosen, which allows for changes in tidal volumes, respiration frequency, and I:E ratio (the ratio between the time allowed for inspiration and expiration). The inspiratory pressure was −30 cm H_2_0 and the expiratory pressure was +2 cm H_2_0 (+5 cm H_2_0 in the first two participants). The I:E ratio was 1:1 in the first 12 participants and 1:1.5 in the last 8 participants. Respiration frequency varied between 8 and 14 (one participant was transiently at a frequency of 6) and was adjusted according to participant comfort and signs of hyperventilation.

### 2.4 Near‐infrared fluorescence imaging

Near‐Infrared fluorescence (NIRF) imaging was used to examine the effects of BCV on peripheral lymphatic function. Indocyanine Green (ICG; Verdye, Diagnostic Green GmbH, Germany//ICG‐pulsion, Nomeco A/S, Denmark) was injected intradermally in the lower limb. The ICG was dissolved in 5 mL of distilled water, of which 0.65 mL was dissolved in 10 mL saline water. Each injection had a volume of 0.1 mL containing 0.03 mg of ICG. Injections on the lower extremity were placed in the 1st and 4th interdigital space on the foot, and behind the medial malleolus. After injection, the area proximal to the injection site was excited by a custom laser with an excitation of 750 nm and excitation surface power of 9 mW/cm (Renkin, 1986). After the excitation, the ICG emits light at 845 nm which is collected by a custom designed complementary metal oxide semiconductor camera that can record wavelengths of >790 nm (Kaer Labs, Nantes, France). Measurements of lymphatic vessel contraction frequency were performed on acquired NIRF imaging sequences.

### 2.5 Strain gauge plethysmography

To investigate the microvascular circulation, strain gauge plethysmography (Hokanson EC6 and E20; Marcom Medical) was used with an analog‐to‐digital converter (ADInstruments, United Kingdom). A venous congestion protocol was carried out over 20 min from 20 to 80 mmHg. A cuff on the lower limb was inflated proximally to the strain gauge, and the pressure was increased by 10 mmHg every 3 min until 80 mmHg was reached. The increase in calf volume due to the venous congestion and extravasation of fluid was recorded continuously by the strain gauge. Strain gauge data was saved in LabChart 8 Software (ADInstruments, United Kingdom) for subsequent analysis. The initial non‐linear increase in calf volume each time the cuff pressure increases is due to increased venous distension, whereas the (potential) subsequent linear increase represents steady state and reflects continuous extravasation of fluid surpassing lymphatic drainage. Taking the slope of the linear time‐volume change (%) during steady state allows for estimation of the capillary filtration rates (μL × 100 mL of tissue^−1^ × min^−1^). The slope was measured over 90 s to minimize sensitivity to artifacts in the measurements.

### 2.6 Study procedure

Study participants were placed in a supine position and ICG was injected on the left lower extremity 15 min prior to NIRF recordings, allowing time for both lymphatic uptake of the ICG and acclimatization of lymphatic contractions in a supine position. Baseline values for strain gauge plethysmography (SGP) were measured on the contralateral lower limb during this acclimatization period. Then baseline values for contraction frequency were recorded using NIRF imaging on the left leg for 12 min. Next, the cuirass was attached, and BCV commenced. NIRF imaging recordings during BCV took place for a total of 2 × 12 min. Subsequently, SGP measurements were repeated under the influence of BCV. External heating of the lower limb was used as a lymphatic stress test (Groenlund et al., 2017; Kelly, Mohanakumar, Telinius, et al., 2020; Mohanakumar et al., 2021) to further investigate the effect of BCV. Heating was accomplished by air‐heating with a Bair Hugger (3 M, Denmark) set at 43°C for 10 min, after which NIRF imaging was recorded for 6 min. The cuirass was then removed and after 20 min of acclimatization, a new baseline NIRF imaging sequence was performed for 6 min, and the heating procedure was repeated followed by another 6 min NIRF imaging sequence (Figure 1).

**FIGURE 1:**
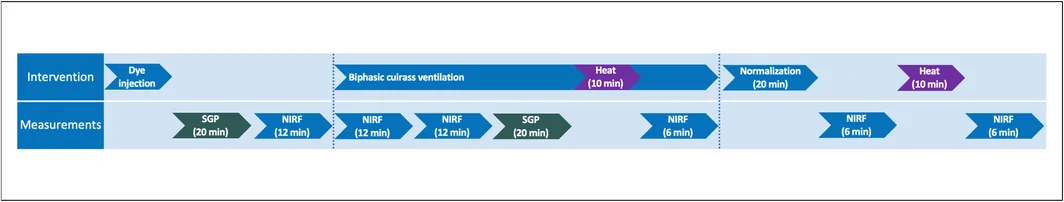
Study procedure timeline. NIRF, near‐infrared fluorescence imaging; SGP, strain gauge plethysmography.

### 2.7 Endpoints

#### 2.7.1 Primary endpoints

Changes in peripheral lymphatic contraction frequency during BCV.

#### 2.7.2 Secondary endpoints

Changes in peripheral lymphatic contraction frequency during external heating. Changes in SGP‐estimated capillary filtration rates during BCV.

### 2.8 Data analysis

#### 2.8.1 Lymphatic contraction frequency

NIRF imaging sequences were analyzed using the image processing program Kaer Analytics (Kaer Labs, Nantes, France). Sequences were blinded before analysis. A contraction is defined as a visible pulsatile movement of a packet of lymph. We report contraction frequency as the number of contractions per minute.

#### 2.8.2 Strain gauge plethysmography

LabChart 8 Software (ADInstruments, United Kingdom) allowed for the measurement of capillary filtration rates. Linear regression correlating the capillary filtration rates to the venous congestion pressure gives us the capillary filtration coefficient (μL × 100 mL of tissue^−1^ × min^−1^ × mmHg^−1^). The intercept of the linear regression line with the X‐axis is defined as the isovolumetric pressure (mmHg), which is the point at which the extravasation of fluid surpasses that of the lymphatic drainage. Negative isovolumetric pressures are excluded from analysis as they are assumed unphysiological.

### 2.9 Statistical analysis

To detect an increase of 30% in peripheral lymphatic contraction frequency, and assuming 80% power and a 95% confidence interval, a total of 17 patients were needed. However, due to potential quality issues with the image sequences, we included 20 participants in total for this study.

#### 2.9.1 Lymphatic contraction frequency

Lymphatic contraction frequency results were analyzed with a paired *t*‐test, and p values were adjusted for multiple testing using the Holm method.

#### 2.9.2 Strain gauge plethysmography

The increase in capillary filtration rate with increasing pressure and the effect of the condition (spontaneous breathing vs. BCV) were analyzed with a repeated measures ANOVA test. Normal distribution of residuals was visually assessed with a Q‐Q plot. Results are reported as F(DFn, DFd) = the *F*‐value, *p*‐values; DFn: degrees of freedom numerator (effect); DFd: degrees of freedom denominator (error). Capillary filtration coefficient and isovolumetric pressure results were analyzed with a paired *t*‐test.

All statistical analysis is performed in R Statistical Software (version 2026.01.0 + 392, R Core Team 2025, https://www.r‐project.org).

## 3 RESULTS

Twenty healthy, young adults were included between August 2023 and June 2024. The results of the first participant were excluded due to quality issues and subsequent changes in the technical approach and study procedure. The mean age of the final 19 participants was 25.5 ± 1.4 years and 9 (47%) were female.

### 3.1 Lymphatic contraction frequency

The mean number of vessels visible for frequency measurements was 3.8 ± 1.3SD. Mean contraction frequency (mean ± SD) was 1.46 ± 0.73/min at baseline, 1.43 ± 0.76/min during BCV, 0.55 ± 0.55 during BCV and heat, 1.51 ± 0.84 at baseline (repeated), and 0.33 ± 0.49 when heated. There was no significant difference in contraction frequency between baseline and BCV (mean difference = 0.03, 95% CI: −0.23–0.29, *p* = 0.81, *n* = 19). Contraction frequency significantly decreased when external heating was applied during BCV (mean difference = 0.88, 95% CI: 0.62–1.13, *p* < 0.0001, *n* = 19). Contraction frequency was likewise significantly decreased between repeated baseline measurements and exposure to heating (mean difference = 1.19, 95% CI: 0.76–1.62, *p* < 0.0001, *n* = 19) (Figure 2).

**FIGURE 2:**
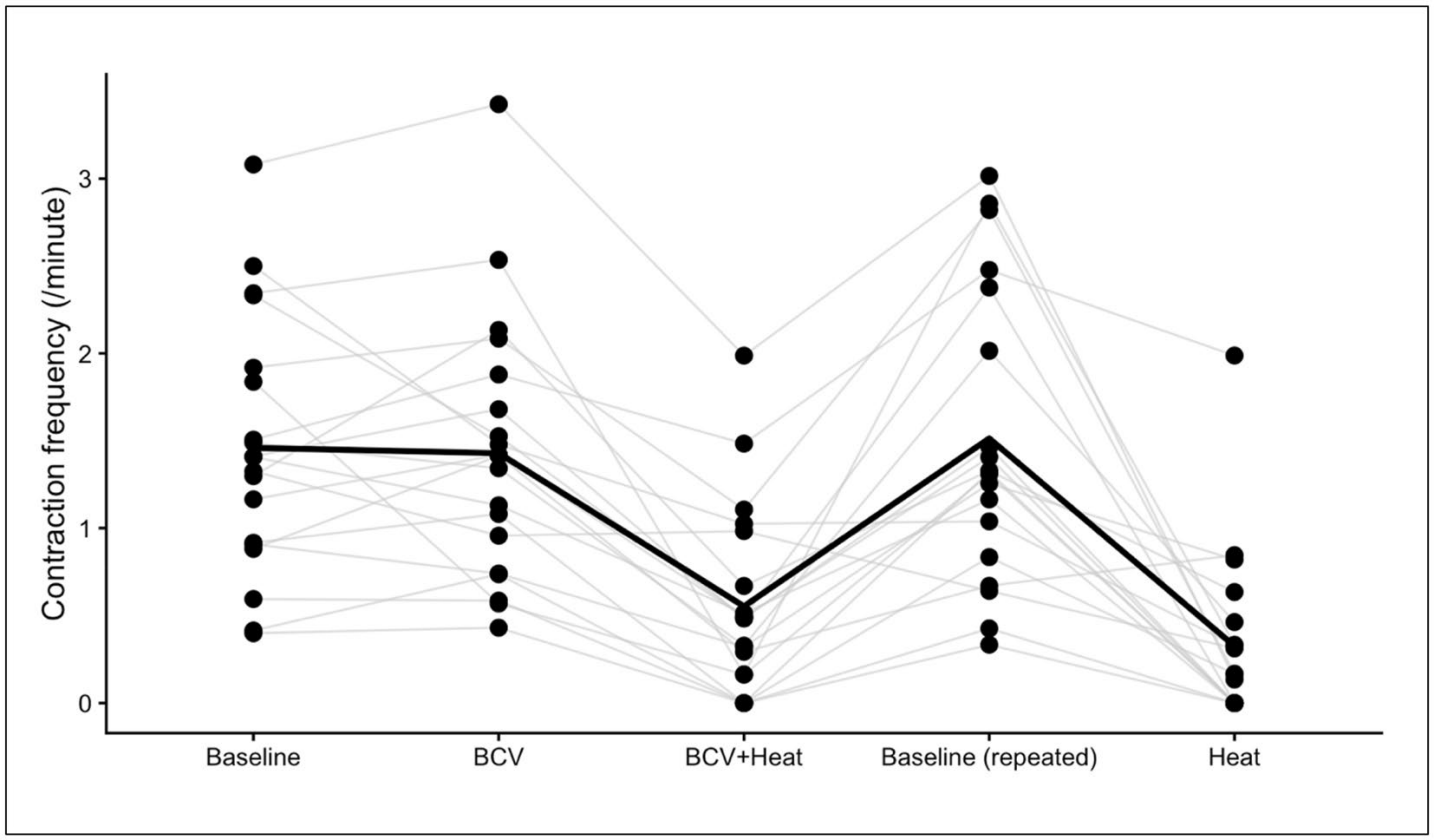
Peripheral lymphatic contraction frequency. Lymphatic contraction frequency during the different conditions. Thick line represents mean contraction frequency. Thin lines are individual measurements. BCV, Biphasic cuirass ventilation.

### 3.2 Strain gauge plethysmography

There was a physiologic linear increase in capillary filtration rate (CFR) with increasing pressure both at baseline and during BCV (*F*(6,108) = 124.50, *p* < 0.0001, *n* = 19). Sphericity was assessed with Mauchly's test, and no correction was necessary. Overall, CFR did not differ significantly between baseline and BCV across all 7 pressure levels (*F*(1,18) = 1.13, *p* = 0.30, *n* = 19) (Figure 3). This was consistent with the isovolumetric pressure, which showed no difference between baseline and BCV (mean difference = −2.61, 95% CI: −8.92–3.71, *p* = 0.39, *n* = 15) (Figure 4). Likewise, there was no significant difference in the capillary filtration coefficient between baseline and BCV (mean difference = 1.21 × 10^−4^, 95% CI: −4,87 × 10^−4^ – 7.29 × 10^−4^, *p* = 0.68, *n* = 19) (Figure 5).

**FIGURE 3:**
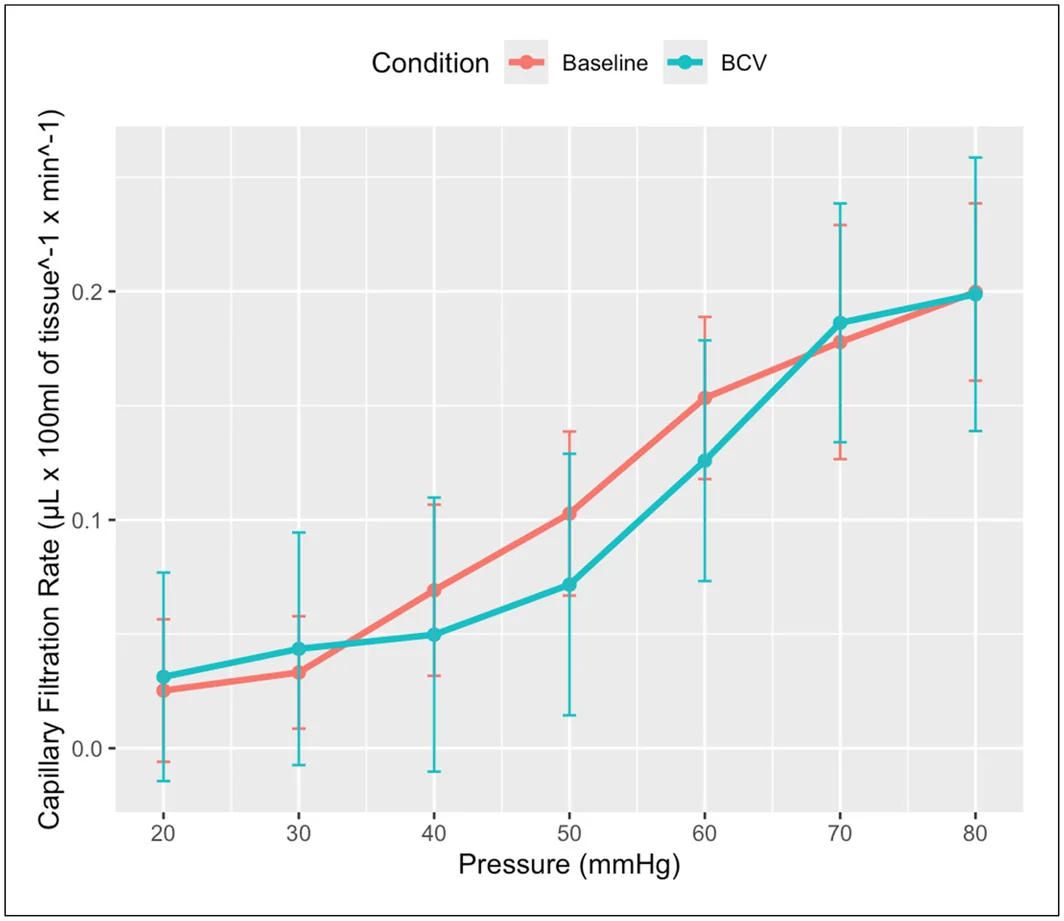
Capillary filtration rate. Capillary filtration rates are estimated with strain gauge plethysmography at 10 mmHg pressure steps during venous congestion protocol. Capillary filtration rates did not differ between baseline and BCV (*F*(1,18) = 1.13, *p* = 0.30, *n* = 19). BCV, Biphasic cuirass ventilation.

**FIGURE 4:**
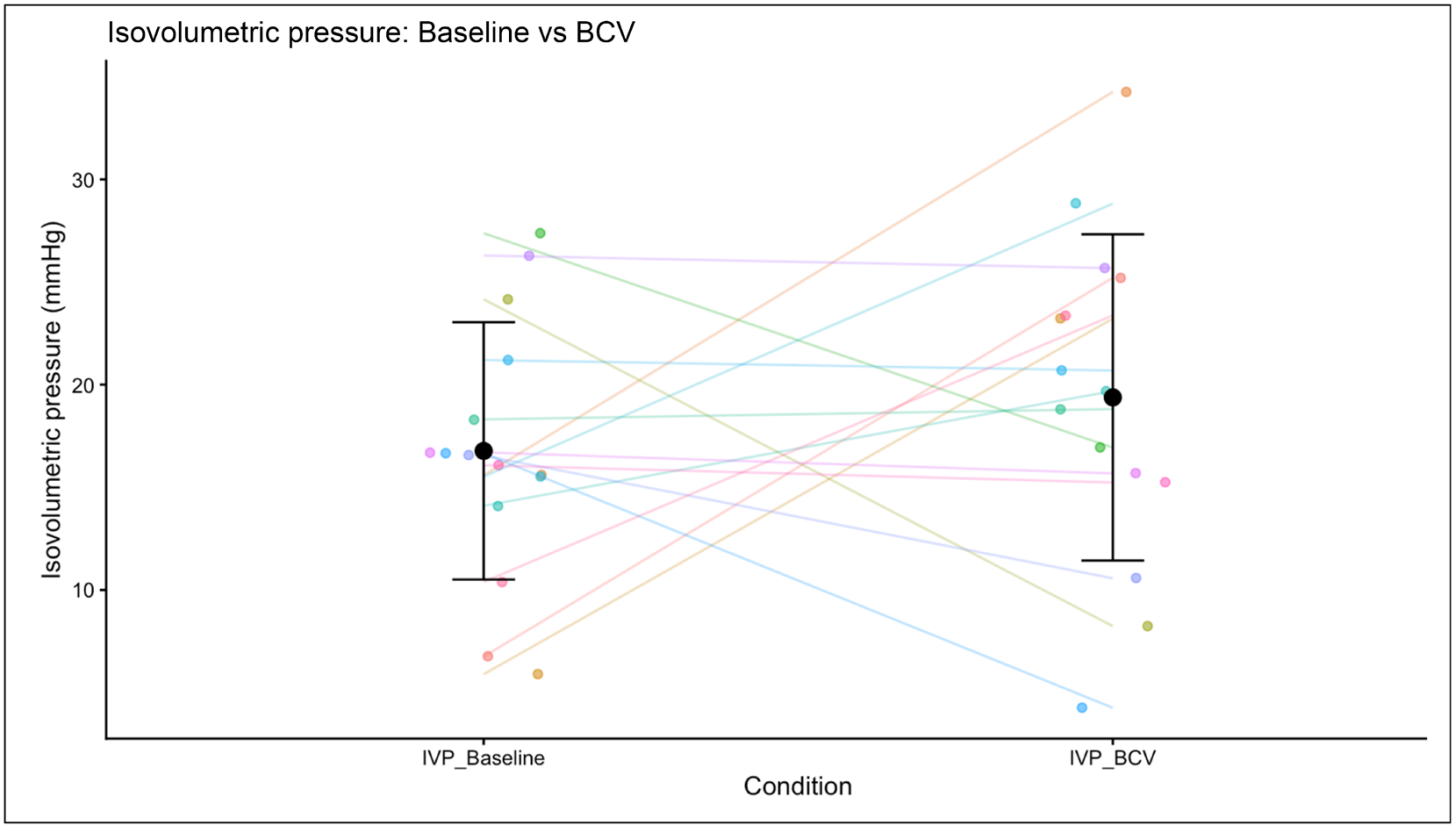
Isovolumetric pressure. Isovolumetric pressures at baseline and during BCV. Isovolumetric pressures represent the highest pressure at which extravasation of fluid and lymphatic drainage is equal. The mean isovolumetric pressure (mean + SD) was 16.77 ± 6.27 (*n* = 15) at baseline and 19.38 ± 7.95 during BCV (*n* = 15), and the difference was not significant (mean difference = −2.61, 95% CI: −8.92–3.71, *p* = 0.39, *n* = 15). BCV, biphasic cuirass ventilation.

**FIGURE 5:**
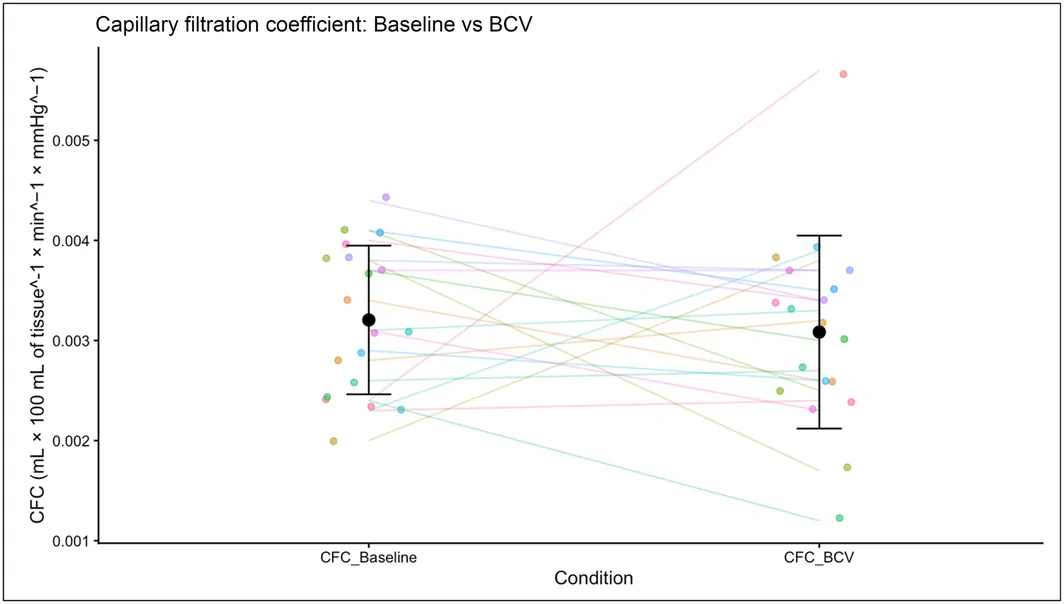
Capillary filtration coefficient. The mean capillary filtration coefficient (mean + SD) was 3.21 × 10^−3^ ± 7.43 × 10^−4^ at baseline and 3.08 × 10^−3^ ± 9.64 × 10^^−4^ during BCV, and the difference was not significant (mean difference = 1.21 × 10^−4^, 95% CI: −4,87 × 10^−4^ – 7.29 × 10^−4^, *p* = 0.68, *n* = 19). BCV, biphasic cuirass ventilation.

## 4 DISCUSSION

In this experimental, interventional study examining the effects of BCV on the peripheral lymphatic function in awake, healthy, young adults, we did not observe any changes in the activity of the peripheral lymphatic system during BCV. The contraction frequency of the peripheral lymphatic vessels remained constant, and likewise the peripheral microvascular circulation demonstrated no changes in capillary filtration rates, isovolumetric pressure, or capillary filtration coefficient.

Our group recently observed decreased peripheral lymphatic activity during PPV in healthy, young adults undergoing anesthesia for elective, corrective jaw surgery (Thorup et al., 2025). This is corroborated by small studies on the central lymphatic system, demonstrating increased pressure (Kelly et al., 2022) and decreased drainage (Khan et al., 2023) of the thoracic duct during PPV that alleviated when PEEP was removed (Kelly et al., 2022; Khan et al., 2023). Animal studies have further shown that not only does PPV decrease the lymphatic drainage function, PPV also increases lymphatic fluid production (Last et al., 1983; Lattuada et al., 2013; Lattuada & Hedenstierna, 2006). The question now remains whether the deleterious effects of PPV on lymphatic function can be mitigated with BCV. While the beneficial hemodynamic effects of BCV have been firmly established (McBride et al., 2012a, 2012b; Okada et al., 2018; Scholz et al., 1997; Shekerdemian et al., 1996; Shekerdemian, Bush, Lincoln, et al., 1997; Shekerdemian, Bush, Shore, et al., 1997; Toida et al., 2007), the effect of BCV on lymphatic function, which too depends on the intrathoracic pressure and central venous pressure, remains largely undescribed in the literature. A single case report describes the resolution of protein‐losing enteropathy and plastic bronchitis in a Fontan patient after BCV. Protein‐losing enteropathy and plastic bronchitis are lymphatic complications typically attributed to a congested lymphatic system due to a chronically elevated afterload (CVP) on the central lymphatic system (Kelly, Mohanakumar, & Hjortdal, 2020). This suggests a beneficial effect of decreasing an elevated afterload for the lymphatic system (Okada et al., 2018). However, this is the first study to investigate the physiologic lymphatic response to BCV. We observed no changes in the peripheral lymphatic activity or the peripheral microvascular circulation during BCV. This likely reflects that healthy, young adults already are in fluid homeostasis without excess interstitial fluid to transport or an elevated afterload for the lymphatic system to drain against. Future studies on BCV during anesthesia are needed to determine if peripheral lymphatic function can be maintained and post‐operative fluid retention and edema avoided.

We also investigated the effect of heat on the lymphatic vessels. Contrary to our previous findings (Groenlund et al., 2017; Kelly, Mohanakumar, Telinius, et al., 2020; Mohanakumar et al., 2021), we found that heat significantly decreased contraction frequency. It is, however, important to note that in the present study, the participants' leg remained in a horizontal position while being heated, whereas in our previous studies, the leg was placed in heated water in a vertical position. As we have also previously demonstrated that contraction frequency increases during gravitational stress (Holm‐Weber et al., 2022), most likely due to increased hydrostatic pressure in the veins, this could be a plausible explanation as to why an increase in contraction frequency was observed during our previous studies and not in this.

## 5 LIMITATIONS

In our study, participants were awake, which presents the challenge of synchronizing spontaneous respiration to that of the cuirass ventilator, and thus questioning whether negative pressures are transmitted to the intrathoracic cavity. However, beneficial hemodynamic effects of cuirass ventilation have previously been demonstrated in awake, healthy volunteers (McBride et al., 2012b).

We are also limited by our methodology. NIRF imaging visualizes the superficial lymphatic vessels, but changes in the deeper vessels remain undetectable. NIRF imaging allows for the measurement of contraction frequency, and although the majority of lymphatic flow is determined by active contractions (Engeset et al., 1977; Scallan et al., 2016), a potential change in passive flow due to the cyclical changes in CVP under BCV would not be detected.

## 6 CONCLUSION

In this experimental, interventional study examining the effects of BCV in healthy, young adults on the peripheral lymphatic function, we observed no changes in peripheral lymphatic activity or peripheral microvascular circulation during BCV. Future studies on the effect of BCV during anesthesia are needed to find the optimal ventilation balance to limit and ameliorate fluid retention and edema in post‐operative and ICU patients requiring ventilation.

## AUTHOR CONTRIBUTIONS

**C. Noah Nilsson:** Conceptualization; data curation; formal analysis; investigation; methodology; project administration. **Lene Thorup:** Conceptualization; data curation; formal analysis; investigation; methodology; project administration. **Benjamin Kelly:** Conceptualization. **Jakob Friis Schmidt:** Conceptualization. **Michael Seltz:** Conceptualization. **Vibeke Hjortdal:** Conceptualization.

## CONFLICT OF INTEREST STATEMENT

Nothing to declare.

## ETHICS STATEMENT

This study was approved by the Scientific Ethics Committees for the Capital Region of Denmark (H‐23031273). All participants gave informed consent before enrollment.

## Data Availability

Analyzed datasets are available from the corresponding author upon reasonable request.

## References

[phy271161-bib-0001] Adair, T. H. , & Guyton, A. C. (1983). Modification of lymph by lymph nodes. II. Effect of increased lymph node venous blood pressure. American Journal of Physiology, 245(4), H616–H622. 10.1152/ajpheart.1983.245.4.H616 6624930

[phy271161-bib-0002] Breslin, J. W. , Yang, Y. , Scallan, J. P. , Sweat, R. S. , Adderley, S. P. , & Murfee, W. L. (2018). Lymphatic vessel network structure and physiology. Comprehensive Physiology, 9(1), 207–299. 10.1002/cphy.c180015 PMC6459625 30549020

[phy271161-bib-0003] Brotons, M. L. , Bolca, C. , Fréchette, E. , & Deslauriers, J. (2012). Anatomy and physiology of the thoracic lymphatic system. Thoracic Surgery Clinics, 22(2), 139–153. 10.1016/j.thorsurg.2011.12.002 22520281

[phy271161-bib-0004] Claure‐Del Granado, R. , & Mehta, R. L. (2016). Fluid overload in the ICU: Evaluation and management. BMC Nephrology, 17(1), 109. 10.1186/s12882-016-0323-6 PMC4970195 27484681

[phy271161-bib-0005] Corp, A. , Thomas, C. , & Adlam, M. (2021). The cardiovascular effects of positive pressure ventilation. BJA Education, 21(6), 202–209. 10.1016/j.bjae.2021.01.002 PMC8134774 34026273

[phy271161-bib-0006] El Zawahry, M. D. , Sayed, N. M. , El‐Awady, H. M. , Abdel‐Latif, A. , & El‐Gindy, M. (1983). A study of the gross, microscopic and functional anatomy of the thoracic duct and the lympho‐venous junction. International Surgery, 68(2), 135–138. 6350208

[phy271161-bib-0007] Engeset, A. , Olszewski, W. , Jaeger, P. M. , Sokolowski, J. , & Theodorsen, L. (1977). Twenty‐four hour variation in flow and composition of leg lymph in normal men. Acta Physiologica Scandinavica, 99(2), 140–148. 10.1111/j.1748-1716.1977.tb10364.x 842370

[phy271161-bib-0008] Groenlund, J. H. , Telinius, N. , Skov, S. N. , & Hjortdal, V. (2017). A validation study of near‐infrared fluorescence imaging of lymphatic vessels in humans. Lymphatic Research and Biology, 15(3), 227–234. 10.1089/lrb.2016.0061 28749720

[phy271161-bib-0009] Holm‐Weber, T. , Kristensen, R. E. , Mohanakumar, S. , & Hjortdal, V. E. (2022). Gravity and lymphodynamics. Physiological Reports, 10(10), e15289. 10.14814/phy2.15289 PMC9117968 35586957

[phy271161-bib-0010] Kelly, B. , Mohanakumar, S. , & Hjortdal, V. E. (2020). Diagnosis and Management of Lymphatic Disorders in congenital heart disease. Current Cardiology Reports, 22(12), 164. 10.1007/s11886-020-01405-y PMC7547563 33037935

[phy271161-bib-0011] Kelly, B. , Mohanakumar, S. , Telinius, N. , Alstrup, M. , & Hjortdal, V. (2020). Function of upper extremity human lymphatics assessed by near‐infrared fluorescence imaging. Lymphatic Research and Biology, 18(3), 226–231. 10.1089/lrb.2019.0041 31526221

[phy271161-bib-0012] Kelly, B. , Smith, C. L. , Saravanan, M. , Dori, Y. , & Hjortdal, V. E. (2022). Spontaneous contractions of the human thoracic duct‐important for securing lymphatic return during positive pressure ventilation? Physiological Reports, 10(10), e15258. 10.14814/phy2.15258 PMC9114659 35581742

[phy271161-bib-0013] Khan, S. , Smith, C. L. , Pinto, E. M. , Taha, D. K. , Gibbs, K. A. , Rosenblatt, S. A. , & Dori, Y. (2023). Effect of positive pressure ventilation on lymphatic flow in pediatric patients. Journal of Perinatology, 43(8), 1079–1081. 10.1038/s41372-022-01563-7 PMC10400414 36434049

[phy271161-bib-0014] Knox, P. , & Pflug, J. J. (1983). The effect of the canine popliteal node on the composition of lymph. The Journal of Physiology, 345, 1–14. 10.1113/jphysiol.1983.sp014961 PMC1193780 6663494

[phy271161-bib-0015] Last, M. , Kurtz, L. , Stein, T. A. , & Wise, L. (1983). Effect of PEEP on the rate of thoracic duct lymph flow and clearance of bacteria from the peritoneal cavity. American Journal of Surgery, 145(1), 126–130. 10.1016/0002-9610(83)90178-2 6336917

[phy271161-bib-0016] Lattuada, M. , Bergquist, M. , Maripuu, E. , & Hedenstierna, G. (2013). Mechanical ventilation worsens abdominal edema and inflammation in porcine endotoxemia. Critical Care, 17(3), R126. 10.1186/cc12801 PMC4056092 23799965

[phy271161-bib-0017] Lattuada, M. , & Hedenstierna, G. (2006). Abdominal lymph flow in an endotoxin sepsis model: Influence of spontaneous breathing and mechanical ventilation. Critical Care Medicine, 34(11), 2792–2798. 10.1097/01.CCM.0000242158.28619.09 16971857

[phy271161-bib-0018] Levick, J. R. , & Michel, C. C. (2010). Microvascular fluid exchange and the revised Starling principle. Cardiovascular Research, 87(2), 198–210. 10.1093/cvr/cvq062 20200043

[phy271161-bib-0019] McBride, W. T. , Ranaldi, G. , Dougherty, M. J. , Siciliano, T. , Trethowan, B. , Elliott, P. , Rice, C. , Scolletta, S. , Giomarelli, P. , Romano, S. M. , & Linton, D. M. (2012a). The hemodynamic and respiratory effects of continuous negative and control‐mode cuirass ventilation in recently extubated cardiac surgery patients: Part 2. Journal of Cardiothoracic and Vascular Anesthesia, 26(5), 873–877. 10.1053/j.jvca.2012.05.021 22795733

[phy271161-bib-0020] McBride, W. T. , Ranaldi, G. , Dougherty, M. J. , Siciliano, T. , Trethowan, B. , Elliott, P. , Rice, C. , Scolletta, S. , Giomarelli, P. , Romano, S. M. , & Linton, D. M. (2012b). The hemodynamic and respiratory effects of cuirass ventilation in healthy volunteers: Part 1. Journal of Cardiothoracic and Vascular Anesthesia, 26(5), 868–872. 10.1053/j.jvca.2012.05.009 22795171

[phy271161-bib-0021] Messmer, A. S. , Zingg, C. , Müller, M. , Gerber, J. L. , Schefold, J. C. , & Pfortmueller, C. A. (2020). Fluid overload and mortality in adult critical care patients‐a systematic review and meta‐analysis of observational studies. Critical Care Medicine, 48(12), 1862–1870. 10.1097/CCM.0000000000004617 33009098

[phy271161-bib-0022] Mohanakumar, S. , Kelly, B. , Turquetto, A. L. R. , Alstrup, M. , Amato, L. P. , Barnabe, M. S. R. , Silveira, J. B. D. , Amaral, F. , Manso, P. H. , Jatene, M. B. , & Hjortdal, V. E. (2021). Functional lymphatic reserve capacity is depressed in patients with a Fontan circulation. Physiological Reports, 9(11), e14862. 10.14814/phy2.14862 PMC8165731 34057301

[phy271161-bib-0023] Okada, S. , Muneuchi, J. , Nagatomo, Y. , Nonaka, K. , Iida, C. , Shirouzu, H. , Matsuoka, R. , Watanabe, M. , & Joo, K. (2018). Successful treatment of protein‐losing enteropathy and plastic bronchitis by biphasic cuirass ventilation in a patient with failing Fontan circulation. International Heart Journal, 59(4), 873–876. 10.1536/ihj.17-292 29794380

[phy271161-bib-0200] R Core Team (2025). R: A Language and Environment for Statistical Computing. R Foundation for Statistical Computing, Vienna, Austria. https://www.R‐project.org/.

[phy271161-bib-0024] Renkin, E. M. (1986). Some consequences of capillary permeability to macromolecules: Starling’s hypothesis reconsidered. American Journal of Physiology, 250(5 Pt 2), H706–H710. 10.1152/ajpheart.1986.250.5.H706 3706547

[phy271161-bib-0025] Scallan, J. P. , Zawieja, S. D. , Castorena‐Gonzalez, J. A. , & Davis, M. J. (2016). Lymphatic pumping: Mechanics, mechanisms and malfunction. The Journal of Physiology, 594(20), 5749–5768. 10.1113/JP272088 PMC5063934 27219461

[phy271161-bib-0026] Scholz, S. E. , Knothe, C. , Thiel, A. , & Hempelmann, G. (1997). Improved oxygen delivery by positive pressure ventilation with continuous negative external chest pressure. The Lancet, 349(9061), 1295–1296. 10.1016/S0140-6736(05)62507-X 9142069

[phy271161-bib-0027] Shekerdemian, L. S. , Bush, A. , Lincoln, C. , Shore, D. F. , Petros, A. J. , & Redington, A. N. (1997). Cardiopulmonary interactions in healthy children and children after simple cardiac surgery: The effects of positive and negative pressure ventilation. Heart, 78(6), 587–593. 10.1136/hrt.78.6.587 PMC1892328 9470877

[phy271161-bib-0028] Shekerdemian, L. S. , Bush, A. , Shore, D. F. , Lincoln, C. , & Redington, A. N. (1997). Cardiopulmonary interactions after Fontan operations. Circulation, 96(11), 3934–3942. 10.1161/01.CIR.96.11.3934 9403618

[phy271161-bib-0029] Shekerdemian, L. S. , Shore, D. F. , Lincoln, C. , Bush, A. , & Redington, A. N. (1996). Negative‐pressure ventilation improves cardiac output after right heart surgery. Circulation, 94(9 Suppl), II49–II55. 8901719

[phy271161-bib-0030] Thorup, L. , Dehn, A. M. , Heiberg, J. , Nisja, E. , Kofod, T. , Kelly, B. , & Hjortdal, V. E. (2025). Reduced lymphatic activity during positive pressure ventilation and general anesthesia‐a cause of postoperative edema? Acta Anaesthesiologica Scandinavica, 69(6), e70066. 10.1111/aas.70066 PMC12141648 40474334

[phy271161-bib-0031] Toida, C. , Shime, N. , Itoi, T. , & Yamagishi, M. (2007). Recovery from Fontan circulation failure by application of continuous negative extrathoracic pressure. Journal of Anesthesia, 21(2), 282–284. 10.1007/s00540-007-0497-y 17458662

[phy271161-bib-0032] Umbrello, M. , Cereghini, S. , & Muttini, S. (2023). Respiratory variations of central venous pressure as indices of pleural pressure swings: A narrative review. Diagnostics, 13(6), 1022. 10.3390/diagnostics13061022 PMC10047347 36980329

